# Swallowtail Butterflies Use Multiple Visual Cues to Select Oviposition Sites

**DOI:** 10.3390/insects12111047

**Published:** 2021-11-22

**Authors:** Hiromi Nagaya, Finlay J. Stewart, Michiyo Kinoshita

**Affiliations:** Laboratory of Neuroethology, SOKENDAI (The Graduate University for Advanced Studies), Shonan Village, Hayama 240-0193, Japan; hiromi2407n@gmail.com (H.N.); finlaystewart4@gmail.com (F.J.S.)

**Keywords:** oviposition, vision, butterfly, behavior, leaf

## Abstract

**Simple Summary:**

Butterflies must not only identify host plants on which to lay their eggs—which they achieve using chemical cues—but also select suitable leaves on that plant that will support the growth of their larval offspring. Here, we asked whether swallowtail butterflies lay eggs on particular leaves of a *Citrus* tree and, if so, which cues they use to select the leaves. We first observed that butterflies indeed select just a few leaves on which to lay eggs. These leaf preferences were observed across many individuals, implying that they were not idiosyncratic, and the butterflies descended directly towards the leaves from some distance, suggesting that they were using visual rather than chemical cues. We then investigated which visual cues are used by the butterflies, and found that the number of eggs laid upon a leaf was correlated with its height on the tree, flatness, green reflectance, brightness, and degree of polarization. These five features may be important both for selecting young leaves and those which are situated well for egg-laying. An outstanding question for future study is how visual and chemical cues interact in this context.

**Abstract:**

Flower-foraging Japanese yellow swallowtail butterflies, *Papilio* *xuthus*, exhibit sophisticated visual abilities. When ovipositing, females presumably attempt to select suitable leaves to support the growth of their larval offspring. We first established that butterflies indeed select particular leaves on which to lay eggs; when presented with a single *Citrus* tree, butterflies significantly favored two out of 102 leaves for oviposition. These preferences were observed across many individuals, implying that they were not merely idiosyncratic, but rather based on properties of the leaves in question. Because the butterflies descended towards the leaves rather directly from a distance, we hypothesized that they base their selection on visual cues. We measured five morphological properties (height, orientation, flatness, roundness, and size) and four reflective features (green reflectance, brightness, and degree and angle of linear polarization). We found that the number of eggs laid upon a leaf was positively correlated with its height, flatness, green reflectance, and brightness, and negatively correlated with its degree of polarization, indicating that these features may serve as cues for leaf selection. Considering that other studies report ovipositing butterflies’ preference for green color and horizontally polarized light, butterflies likely use multiple visual features to select egg-laying sites on the host plant.

## 1. Introduction

Many animals rely on vision to identify suitable targets for various behaviors including foraging, mating, and habitat selection. The visual abilities of flower foraging bee species have been particularly well studied [1,2]. Bees can learn to associate the color and shape of flowers with different quantities and qualities of nectar or pollen, and make foraging choices on this basis [3,4,5].

Like bees, many butterfly species feed from flowers. Diurnal butterfly species depend heavily on vision to detect flowers [6,7,8,9,10,11,12]. One such species, the Japanese swallowtail butterfly *Papilio xuthus,* can discriminate between artificial targets on the basis of color, brightness, and the *e*-vector angle of polarized light [13]. *Papilio*’s compound eye consists of three types of ommatidia. Each ommatidial type contains a particular arrangement of spectral receptors among six classes (UV, violet, blue, green, red, and broad-band receptors). In addition, each photoreceptor is sensitive to a certain *e*-vector angle of polarized light (polarization sensitivity) [14]. This complex retinal organization facilitates acute color discrimination. In addition, recent studies have demonstrated that chromatic and polarization contrast contribute not only to visual object detection, but also to *Papilio*’s motion vision [15,16].

Oviposition is clearly an evolutionarily important behavior for female butterflies. They identify appropriate host plants using their chemical senses, i.e., olfaction and gustation [17,18,19,20]. Pierid butterflies (Pieridae, Lepidoptera) preferentially select leaves of plants grown in nutrient-rich soil [21,22], which promotes the development of the larvae. Female *Melitaea cinxia* not only detect iridoid glycosides as a cue of the host plant, but also the pre-oviposition level of iridoid glycosides aucubin on the host plant [23]. These examples indicate the diverse roles played by contact chemosensory systems in butterfly species.

As in flower foraging, vision also plays a role in egg-laying. *Papilio aegeus* prefers greenish targets for oviposition, as do *Pieris* butterflies [8,24,25,26], indicating that butterflies might use greenness to distinguish young leaves at a distance. *P. aegeus* also prefers horizontally polarized light, which could help them to identify flat, horizontally-oriented leaves that afford easy landings [27,28]. Nymphalid checkerspot butterflies select leaves according to visual cues such as their size, shape, and orientation [23]. Thus, it appears that several visual cues can contribute to the animal’s evaluation of leaves as potential oviposition targets. However, laboratory studies typically use artificial stimuli, raising questions about the ecological relevance of these preferences in the wild.

In this study, we observed *Papilio* with the aim of determining whether they select particular leaves of a host *Citrus* tree. We then investigated whether they use visual cues to select leaves on a host plant and, if so, which visual features are salient. We demonstrated that *P. xuthus* do indeed favor particular leaves for oviposition, and that these preferences are similar across individuals. We found significant correlations between the number of eggs laid on each leaf and five of their visual features: height, flatness, green reflectance, brightness, and the degree to which the light they reflect is polarized. We determined that ovipositing *P. xuthus* preferred easily accessible, flat, bright leaves with matte surfaces under our experimental conditions.

## 2. Material and Methods

### 2.1. Animals

We used summer-form female adult *Papilio xuthus* (Papilionidae, Lepidoptera, Linnaeus) butterflies. As larvae, we fed them fresh *Citrus* leaves and raised them under a 14/10 h light/dark regime at 25–28 °C. Following pupation, we hand-mated newly emerged females with males that had emerged a few days previously. We kept mated females individually in dimly-lit boxes to keep them calm and prevent any damage to their wings before the experiment. From the second day after emergence, we fed the females sucrose solution (10%) daily on white paper until they were sated. For our experiments, we used females from their third day post-emergence.

### 2.2. Behavioral Experiment

To effectively elicit oviposition behavior in *Papilio xuthus*, we informally tested various trees, artificial visual stimuli, lighting conditions, cage sizes, and treatments of mated females. We found that butterflies laid eggs most frequently and reliably in a relatively small cage, on a potted tree rather than artificial targets.

### 2.3. Experimental Setup

**Figure 1:**
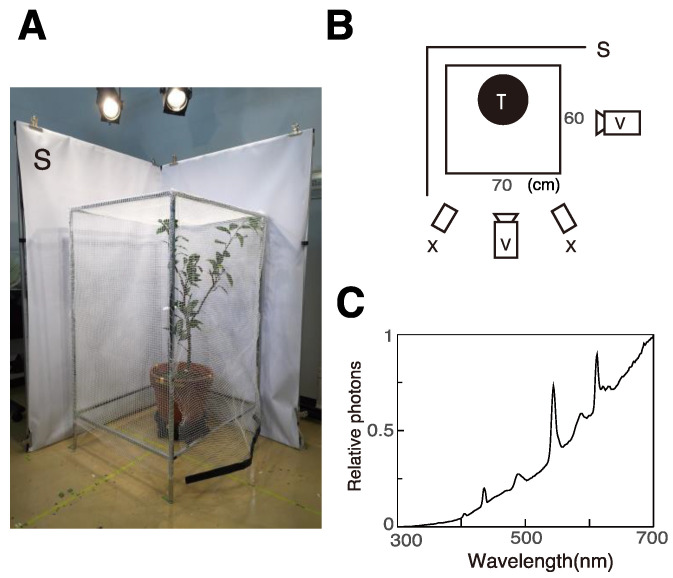
Experimental setup. (**A**) Photograph of the setup with a small cage. (**B**) Top view of the setup. (**C**) Irradiation spectrum of illumination. S: Screen, V: video camera, X: Solar lamp.

We observed one female laying eggs on a potted tree, *Citurus unshiu,* in a small cage (105 × 60 × 70 cm, Figure 1A) in each experiment. We used two tripod-mounted video cameras (Panasonic HC-X920M, Japan, Figure 1B) to record the animal’s behavior from roughly orthogonal angles. White screens were placed outside the two opposite walls of the cage to provide a uniform backdrop for the footage.

**Figure 2:**
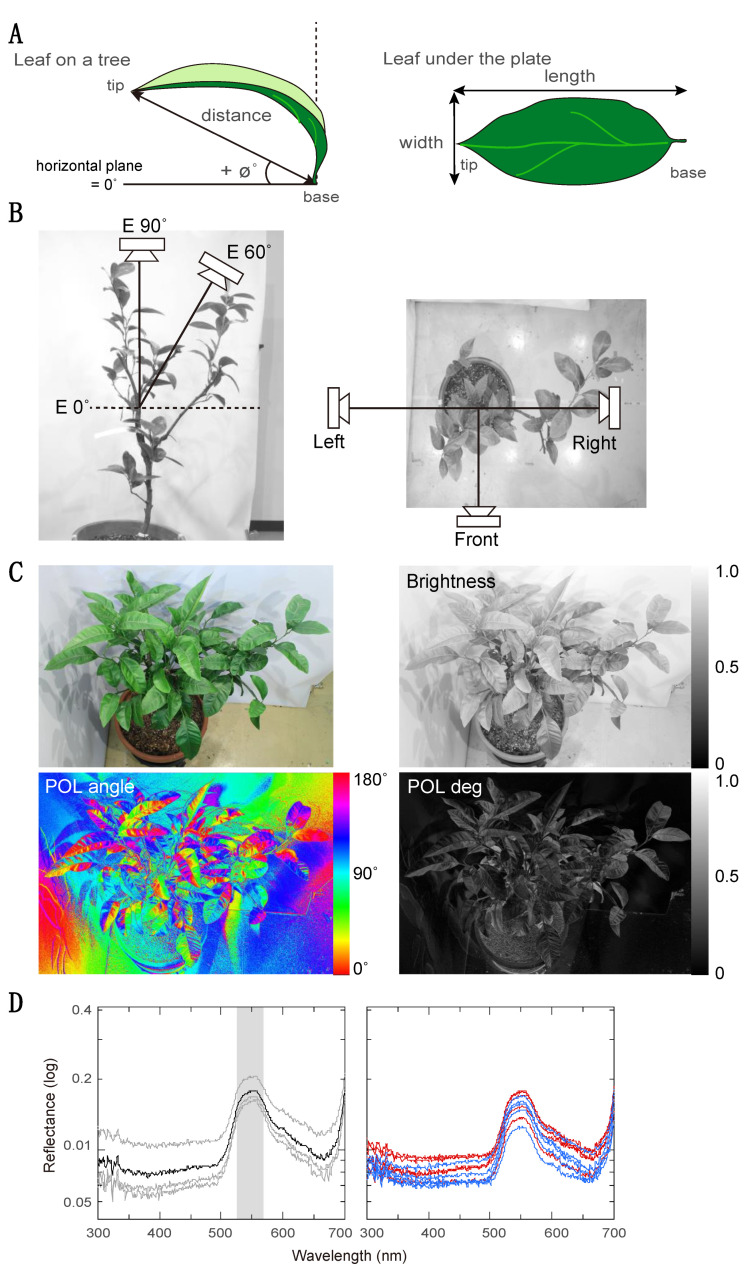
Morphological and reflective features of leaves. (**A**) Measurement points for orientation, flatness, and roundness. The orientation of the leaf and the distance between base and tip were measured while the leaf was on the tree (upper), then the leaf was removed from the tree and placed flat under a transparent sheet in order to measure its length and width (lower). Flatness is the ratio of distance to length; roundness is the ratio of width to length. (**B**) Positions of a camera with a polarizer for measuring brightness and polarized light information. We took photos from four different positions: one directly above the tree at 90° elevation (left), and three from the front, left, and right sides of the tree (right) at 60° elevation (left). (**C**) Grayscale or false color images showing brightness (upper right), e-vector angle (lower left) and degree of polarization (upper right). The upper left image is an ordinary photograph with no polarizer. Scales are shown at the right side of each image; brightness and polarization degree are in arbitrary units. (**D**) Reflectance spectra of a leaf. We measured reflectance spectra at three regions on a leaf (thin lines) and took their mean (thick line), shown in the left graph. Reflectance of the five favored (red lines) and five unfavored leaves (blue lines) are presented in the right graph. Green reflectance is defined as the integral of the mean spectrum from 530 nm to 570 nm (shaded area).

We illuminated the cage with four fluorescence tubes, ten halogen lamps, and two solar lamps (XC-100BF, SERIC, Japan, Figure 2B). Light intensity was approximately 7000 and 3000 lux at the top and bottom of the cage, respectively. Irradiation spectra were measured as reflection from the surface of a white MgO disk using a spectrometer (HSU-100S, Asahi Spectra, Japan, Figure 1C). Ambient temperature during experiments was between 30 and 35 °C.

### 2.4. Procedure of Behavioral Experiment

We carried out the behavioral experiments in September and October 2015. Before each experiment, we allowed each butterfly to feed on sucrose solution (10%), and then returned it to its box for three hours. We released a single female into the cage containing a single *Citrus* tree and observed its oviposition behavior. Each tested tree bore approximately one hundred leaves. The observation lasted for fifteen minutes from the time the butterfly laid its first egg. After the experiment, we assigned ID numbers to leaves of the tree, counted the number of eggs on each leaf, and gently removed all eggs manually before conducting the next observation with a different individual. If a butterfly did not lay any eggs within ten minutes of being released, we abandoned the observation. We were keen to use each tree for as short a time as possible, as the condition of its leaves cannot be assumed to stay constant over several days, particularly under the intense heat and light of our laboratory setting.

We video recorded the entire session, using an audio cue to allow the two videos to be synchronized. Thus, we could analyze the footage offline to reconstruct 3D flight trajectories in a manner similar to that described in Stewart et al. (2015, See details in Supplementary S1). In addition, an experimenter observed the animal’s behavior and verbally recorded instances of leaf drumming, abdomen curling, and oviposition.

### 2.5. Measurement of Visual Features

To identify which visual cues female *Papilio* used for selecting oviposition sites, we measured several features of leaves from five trees on which more than three individuals each laid more than twenty eggs. For each tree, we identified the five leaves which received the most eggs. We term these “favored” leaves. We also identified five “unfavored” leaves, which received few or no eggs despite being in similar positions on the tree to the favored ones and being of apparently similar quality (albeit to a human eye).

We measured five morphological features of these leaves: height, pitch orientation, flatness, roundness, and size (Figure 2A). We define the height of a leaf as the vertical distance from its tip to the floor. We define pitch orientation as the angle of a straight line from the base to the tip of the leaf, relative to the horizontal plane. A positive value of the pitch orientation means that the tip is higher than the basal point, whereas a negative value indicates that the tip is lower than the basal point. Flatness is the ratio of the 3D distance from the base to the tip of the leaf on the tree, to the length of the leaf when laid flat under a glass plate; thus, a value of 1 denotes a leaf that is perfectly straight along its long axis. Roundness is the ratio of the width (at the widest point) to the length of the leaf when laid flat. Size is the area of the flattened leaf, measured by counting the number of squares it covers on 1 cm grid paper. In total, we measured these features of fifty leaves taken from five trees.

We measured four reflective properties of the leaves: polarization degree (POL-degree), polarization angle (POL-angle), brightness, and green reflectance. We photographed the tree from four positions: directly overhead, and from an elevation of 60° at three azimuthal positions 90° apart (Figure 2B), which approximately corresponded to the view of butterflies based on their flight area in the cage. To measure brightness and polarization properties, we took a photograph with a digital camera (Canon EOS M, Tokyo, Japan) through a linear polarizer (Kenko, PL) oriented at three angles (0°, i.e., horizontal, 60°, and 120°) at each camera position. We then converted the three images (polarizer angle of 0°, 60°, and 120°) to grayscale and assigned these grayscale images to the three color channels to produce a single false color RGB image. We converted this RGB image to HSB (hue, saturation, brightness) format. In this representation, the H channel corresponds to angle of polarization (Figure 2C bottom left), S to degree of polarization (Figure 2C bottom right), and B to brightness (Figure 2C upper right). In the obtained channels, each pixel has a scalar value between 0 and 255 representing each feature. For brightness and degree of polarization, we mapped these values of 0 to 255 to the range of 0 to 1; note that these measurements are thus relative (i.e., in arbitrary units) rather than absolute. For POL-angle, we similarly mapped the value of 0 to 255 to the range of 0 to 180°, and then applied a cosine function to convert the circular metric into a linear one, i.e., a horizontal vs. vertical continuum from 1 to -1. To measure the properties of each leaf, we selected the pixels corresponding to that leaf in the image (note that it may be partially occluded by other leaves) and took the mean value. Unlike our measure of green reflectance, which was concerned with the material properties of each leaf, these measurements aimed to describe the leaf’s appearance in situ on the tree, under the lighting conditions of the experiment. For brightness and POL degree, we obtained the mean value from the three images taken at same elevation (E60). However, we treated POL angle separately for each azimuthal position as averaging would not be meaningful in this case, because the POL angle of reflected light depends on the observer’s orientation. We performed all analysis of the photographs using ImageJ.

To measure “green” reflectance, we removed the leaf from the tree. We then immediately obtained the mean reflectance spectrum of each leaf from spectra sampled at three regions (tip, middle, and base, 2 cm diameter each) of the leaf using a spectrometer (Figure 2D). We integrated the reflectance in the 530–570 nm band to quantify green reflectance, which we express as a relative quantity, normalized to the maximum value obtained across all leaves.

We used each tree for just a few days to minimize changes in its appearance between observations. We measured all the visual features of leaves on the final day of using each tree. We first took pictures to measure polarization and brightness, then measured the heights of the leaves, and finally cut the leaves from the tree to measure the remaining morphological features and reflectance spectra.

### 2.6. Statistical Analysis

We applied Wilcoxon signed-rank tests [29] in R to determine whether particular leaves of each tree were significantly favored for oviposition over the rest. We then calculated the proportion of each individual’s total eggs that were laid on each leaf, and averaged this measure across individuals. We then compared these values to those expected by chance, i.e., assuming eggs were randomly distributed across leaves. For this analysis, we conservatively only included those leaves which received at least one egg, as we know that these ones were possible for the butterflies to access.

We used Wilcoxon’s rank sum test [29] to determine whether the various measures of leaf features differed between the favored and unfavored groups (50 leaves from the five trees in total). As this distinction between “favored” and “unfavored” could be argued to be artificially binary, we also investigated whether the features of the ten sampled leaves on each tree correlated with the proportion of eggs each one received. To do this, we ranked the ten leaves from a single tree on each of the visual features, and on the number of eggs they received (tied ranks were averaged), and then calculated the Pearson’s correlation coefficient [29] for the ranks. We visualized these relationships using bubble charts, where the size of the circle denotes the number of observations. R was used for both Wilcoxon’s rank sum test and the Pearson’s correlation coefficient for the ranks.

## 3. Results

### 3.1. Oviposition Behavior and Leaf Selection

**Figure 3:**
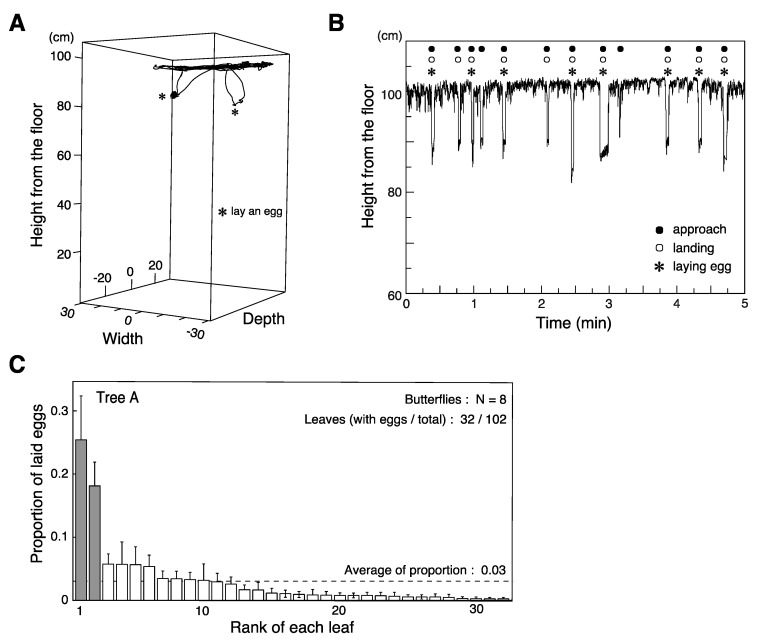
Observation of ovipositing behavior. (**A**) 3D flight trajectory of a butterfly over three min. * denotes instances where it laid eggs. (**B**) Flight altitude during ovipositing behavior. The animal flies close to the ceiling, intermittently making distinct descents to approach leaves. (**C**) Mean proportions of eggs laid on leaves of a single tree by eight individuals. Two leaves (gray bars) received a significantly higher proportion than the chance baseline, shown as a dashed line.

On five trees (Tree A–E) of the eight we tested, at least three butterflies each laid more than twenty eggs. We analyzed in detail the oviposition behavior of the eight individuals tested on Tree A (Figure 3A,B). When investigating which visual features were associated with oviposition, we pooled data from 23 individuals across the five trees (Figure 3C, Supplementary Figure S2).

The ovipositing behavior of *P. xuthus* consists of five steps: 1. approaching a leaf, 2. landing, 3. drumming the leaf with forelegs, 4. curling the abdomen until the ovipositor touches the surface of the leaf, and 5. depositing an egg [30]. Butterflies are initally attracted to the vicinity of the host plant by olfaction. They use visual cues to locate the host plant, then evaluate leaves and select one to approach. After landing on the leaf, they use contact chemosensation to confirm its suitability. Visual cues contribute to the initial steps of this sequence. In our experimental condition, the process typically took a few seconds. In most cases, the egg was laid on the upper surface of the leaf, as it typically is in the natural habitat. We analyzed details of ovipositing behavior in eight butterflies tested on the same tree by combining our flight trajectory data with the verbal annotations recorded during the experiment (Figure 3A,B). The butterflies flew around roughly 5 cm below the ceiling of the cage (i.e., roughly 10–15 cm above the leaves on the tree; Figure 3B, Table 1) and frequently descended towards a targeted leaf. After landing on the leaf, the butterfly would drum the leaf with its forelegs, curl its abdomen, and lay an egg. It would then take off and return to near the ceiling of the cage before repeating the process. The butterflies approached leaves 78.5 ± 8.4 (mean ± s.e.) times during the 15 min session. They performed drumming and curling on the leaf after 93.3% ± 1.6 of approaches, but only laid an egg in 60.5% ± 4.4 of approaches.

Different individuals would tend to select the same few leaves among the roughly one hundred on the tree (average number of leaves 103.8 ± 6.6, Figure 3C, Supplementary Figure S2). Figure 3C shows the proportion of eggs laid by eight individuals on each leaf of tree A. We found eggs on 32 of the 102 leaves on the tree in total (plus one egg laid on a branch). Among these 33 locations, two leaves received significantly higher proportions of eggs (25.4% and ~18.1%) than chance would predict, i.e., 1/33 = 3.0% (Figure 3C). A similar pattern of females disproportionately favoring a few leaves was also seen on the other trees, though this tendency was not statistically significant, likely due to the small numbers of individuals involved (Supplementary Figure S2) and/or our conservative definition of the chance baseline (see Methods).

### 3.2. Visual Features of Leaves

**Figure 4:**
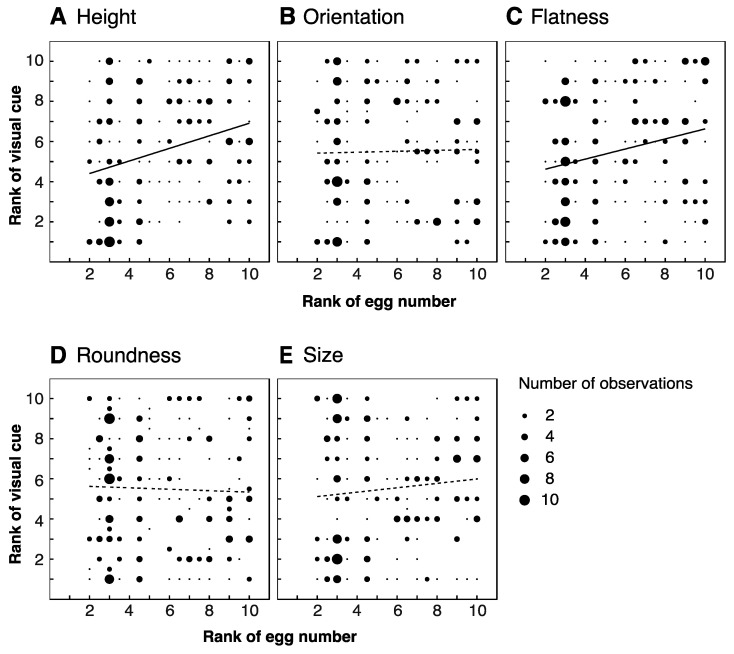
Correlation between morphological features of leaves and number of eggs laid. Height ((**A**): r = 0.285, *p* ≤ 0.01), orientation ((**B**): r = 0.021, *p* = 0.751), flatness ((**C**): r = 0.229, *p* < 0.01), roundness ((**D**): r = −0.033, *p* = 0.620) and size ((**E**): r = 0.998, *p* = 0.131). Thick regression lines denote significant correlations, dashed lines indicate non-significant correlations. Data is based on 50 leaves from five trees.

**Figure 5:**
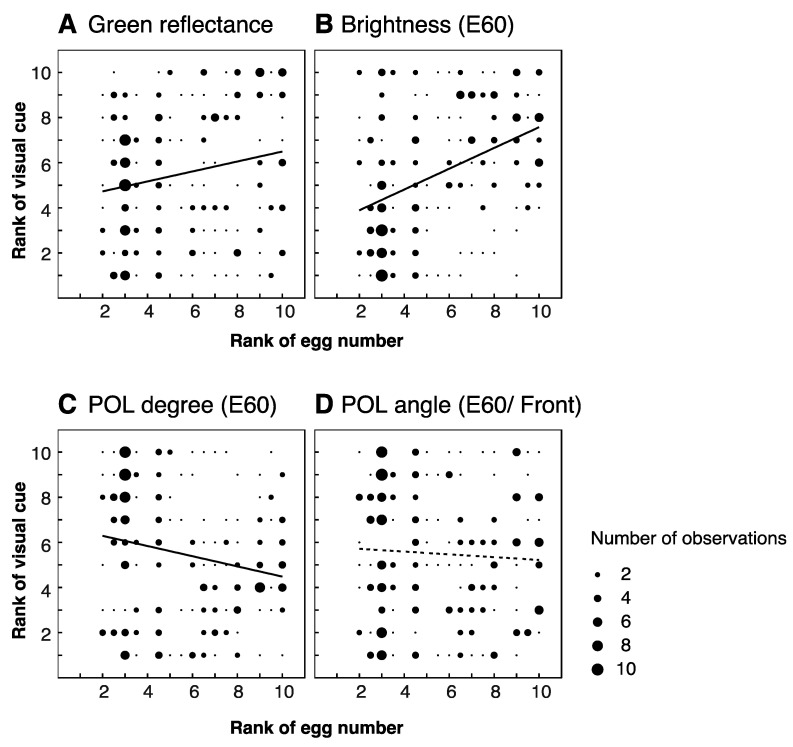
Correlation between reflective features of leaves and proportion of eggs laid. Reflective features: green reflectance ((**A**): r = 0.201, *p* < 0.01), brightness ((**B**): r = 0.419, *p* < 0.01), polarization degree (viewed at 60° elevation, (**C**): r = −0.206 *p* < 0.01), polarization angle (viewed from front at 60° elevation, (**D**): r = −0.056 *p* = 0.397). Thick regression lines denote significant correlations, dashed lines indicate non-significant correlations. Data is based on 50 leaves from 5 trees.

We analyzed the visual features of ten leaves (five favored and five unfavored leaves) from each of the five trees. We did not find significant differences between favored and unfavored leaves in any of the morphological or visual properties that we investigated (Table 1). However, we did identify several significant correlations between these features and the proportions of eggs laid on the leaves. We found positive correlations between number of eggs laid and both leaf height and flatness (r = 0.285, *p* < 0.01 for height; r = 0.229, *p* < 0.01 for flatness; Figure 4A,C), but neither orientation, roundness, nor size were correlated with number of eggs (r = 0.021, −0.033, 0.998; *p* = 0.751, 0.620, 0.131 respectively; Figure 4B,D,E). Both green reflectance and brightness correlated positively with number of eggs (r = 0.201, *p* < 0.01; r = 0.419, *p* < 0.01; Figure 5A,B respectively, also Supplementary Figure S3A). A negative correlation was identified for polarization degree, such that leaves reflecting more weakly polarized light received more eggs (r = −0.206, *p* < 0.01, Figure 5C, Supplementary Figure S3B). We found no significant correlation for polarization angle (r = −0.056, *p* = 0.397, Figure 5D, Supplementary Figure S3C,D).

## 4. Discussion

### 4.1. Leaf Selection for Oviposition and Visual Cues

Ovipositing *Papilio xuthus* chose specific leaves on which to repeatedly lay eggs, although the tendency was only significant for one of the five trees we used (Figure 3C). This could potentially be explained by individuals learning the position and/or appearance of an arbitrarily selected leaf and then simply returning to it. *Battus philenor*, for instance, can be trained to lay eggs on targets of particular colors [31]. However, these accounts do not explain our observation that different butterflies selected the same leaves. Therefore, each individual of *P. xuthus* presumably uses similar criteria (i.e. innate preference) to select leaves for oviposition.

It is well known that butterflies use their senses of olfaction and taste to identify host plants. However, we observed that butterflies tended to target leaves from a distance of 10 cm or more, and descended rather directly towards them (Figure 3A,B). This flight behavior is qualitatively similar to that of foraging butterflies approaching visual targets presented on the floor of a small cage without any odor cues [15]. Given the turbulent, unpredictable nature of odor plumes (exacerbated by the downwash from the butterfly’s own wings) produced by many leaves in the closed experimental room, it strikes us as implausible that this kind of target selection at range could be achieved using olfactory cues. Clearly, butterflies cannot use contact chemical cues to select leaves before landing. We therefore assume that visual cues play a dominant role in guiding this behavior, although we do not completely reject the possibility that chemical cues also contribute.

In most cases when butterflies landed on leaves, they curled their abdomen and touched their ovipositor to the leaf. These observations indicate that the butterflies accepted the plant as a suitable host based on its taste (as determined by drumming) in our experimental condition. *Papilio protenor* does not curl its abdomen after drumming on leaves if chemical cues are inappropriate or insufficient [32]. We observed curling in the overwhelming majority of cases where the butterfly landed and drummed on a leaf. We therefore assume that in instances where no egg was laid, this was not because the leaf was rejected on the basis of chemical cues, but perhaps because the animal was unable to make a solid mechanical contact with the leaf, or was otherwise not ready to deposit an egg because too little time had elapsed since laying the previous one. We restricted our analysis of visual cues for leaf selection to leaves which had received eggs in order to exclude leaves that the animals rejected because of chemical cues or other non-visual factors.

### 4.2. Correlations between Number of Eggs and Visual Cues

We found the height and flatness of a leaf to be positively correlated with the number of eggs laid upon it (Figure 4A,C). It may be the case that flat leaves at higher locations are simply the easiest for butterflies to reach from where they typically fly (5 cm below the ceiling) and to stably land and/or lay eggs on. We found no such correlation for orientation, roundness, nor size (Figure 4B,D,E). However, it might be that there was insufficient variability to reveal any effect of these features among the leaves of trees we assessed (Table 1). We cannot exclude the possibility that these features would play a role in selection among more heterogeneous leaves, e.g., in early spring when newly grown leaves are present (see below).

Green reflectance and in situ brightness of leaves are highly correlated with the number of eggs they receive (Figure 5A,B). The reflectance spectra (Figure 2D right) of all leaves are quite similar, and indeed they all appear to be a similar shade of green to the human eye (Figure 2C upper left). Thus, it seems that attractive leaves are not greener than unattractive ones, but rather lighter in overall shade. We performed our experiments at the end of summer, when all leaves were at least several months old. The reflectance spectra of the leaves are similar to those of intermediate age leaves in a previous study of *Papilio aegeus* [8]. In this study, *P. aegeus* preferred to lay eggs on young leaves, which were bright green, rather than intermediate or old leaves, which were duller and more yellowish. In fact, *P. xuthus* also strongly prefers to lay eggs on very small young shoots (personal observation), which are light green in color. In our experiment, therefore, butterflies may have been using brightness cues in an attempt to select the youngest leaves, despite no particularly young leaves being present on the trees. As foraging *P. xuthus* can discriminate both color and brightness and flexibly switch between these cues depending on context [13], it is reasonable to suppose that ovipositing butterflies can selectively use either color or brightness to find suitable leaves for oviposition. In addition, butterflies in the wild may, like birds, use UV cues [33], but under our artificial illumination this information would have been absent.

Although *Papilio* butterflies can discriminate between light polarized to different angles [13,27,28], we did not find any correlation between polarization angle and number of eggs laid (Figure 4G). Once again, we cannot exclude the possibility that polarization angle could, in principle, be used to select appropriate leaves; flat, horizontally oriented leaves would tend to reflect horizontally polarized light. Consistent with this notion, ovipositing *P. aegeus* prefer horizontally polarized targets [27].

Polarization degree, on the other hand, was (negatively) correlated with number of eggs laid (Figure 4F). This cue might help to identify target leaves against the background, which has a low degree of polarization (Figure 2D, lower right). Two recent reviews make the point that for invertebrates, polarization degree may represent a more useful signal than polarization angle for the purposes of object detection [34,35]. Because an object’s angle of polarization depends on the relative positions of both the observer and the light source(s), it is not a stable cue for a moving animal. On the other hand, polarization degree information provides more consistent contrast between targets and background [35]. Another, possibly complimentary, potential role for polarization degree is in assessing the quality of leaves. As leaves age, their surface becomes waxier and thus shinier. In other words, younger leaves have a more matte appearance, meaning that they reflect less strongly polarized light.

To reconcile our observation that low polarization degree is attractive with the previous finding that the horizontally polarized light is attractive [27], perhaps it is more appropriate to consider vertically polarized light repellent for oviposition. We have already seen that brightness appears to be an attractive cue. It remains an open question whether polarization information is integrated with the brightness signal to form a single channel, or whether these modalities are perceived independently.

Our intention in this study was to use real *Citrus* trees, albeit under unnaturally constant illumination, rather than artificial stimuli. Consequently, an important caveat in interpreting the above findings is that the various visual features of leaves that we measured are themselves correlated. This being the case, we cannot be certain which features are most salient to the animals, or indeed whether some of them are utilized at all. Furthermore, we cannot exclude the possibility that chemical cues (which may be correlated with visual features) could play an important role not only in host plant identification, but also in the selection of particular leaves. 

### 4.3. Ecological Perspectives

In the natural habitat, butterflies seldom lay an egg on the same leaf twice, thereby dispersing their eggs over many leaves and trees. In the far more restricted environment of our experiments, the butterflies’ oviposition behavior appeared qualitatively normal, except that they repeatedly laid eggs on particular leaves. It seems, therefore, that *P. xuthus* has no specific mechanism preventing laying multiple eggs on an attractive leaf; presumably its behavior simply makes re-encountering a favorable leaf an infrequent occurrence in a natural setting.

Ovipositing butterflies likely evaluate leaves according to a number of criteria, utilizing multiple visual cues. *Pieris rapae* preferentially select leaves with higher nutrient content [21]. Caterpillars of *P. aegeus* develop better on young leaves [8]. These studies indicate that there is an evolutionary pressure for female butterflies to choose high-quality leaves on which to lay eggs. Our findings showed that female butterflies are indeed discerning in their leaf choice, and that several forms of visual information may be involved in this evaluation. Brightness, green reflectance, and polarization degree may indicate the quality and/or age of leaves, while flatness might be more linked to ease of landing. Just as foraging butterflies can switch between using color and brightness cues depending on the presented stimuli [13], ovipositing females may draw upon multiple visual modalities besides other chemical cues to identify the most favorable leaves for their offspring.

## 5. Conclusions

*Papilio* butterflies’ behavior strongly relies on vision. In this study, we investigated the roles of multiple visual cues in guiding the selection of leaves for egg-laying. We found that the number of eggs laid on a leaf was correlated with five visual features, which may use to judge both leaf quality and ease of landing. While our work focused on vision, many previous studies have emphasized the contribution of the chemical senses to oviposition behavior. How visual, olfactory, gustatory, and tactile cues are integrated by butterflies ovipositing in the natural habitat remains an open question for further inquiry.

## Figures and Tables

**Table 1:** Five morphological and four reflective features of favored and unfavored leaves (25 leaves for each type). Mean value (±*SE*) of twenty-five leaves for favored and unfavored leaves. *p* value is obtained by Wilcoxon ranked sum test.

	Morphology				
Leaf Type	Height (cm)	Orientation (−90° < *O* < +90°)	Flatness (0 < *F* < 1)	Roundness (0 < *R* < 1)	Size (cm^2^)
Favored	86.10 ± 1.11	2.80 ± 4.47	0.93 ± 0.01	0.50 ± 0.02	49.58 ± 3.37
Unfavored	82.73 ± 2.10	2.52 ± 5.40	0.90 ± 0.01	0.46 ± 0.02	47.57 ± 4.40
*p* value	0.37	0.96	0.19	0.37	0.59
	**Reflected Light**				
**Leaf Type**	**Rel. Green Reflectance** **(0 < *G* < 1)**	**Brightness (E60)** **(0 < *B* < 1)**	**POL Angle (E60/Front)** **(Degree)**	**POL Degree (E60)** **(0 < *D* < 1)**	
Favored	0.74 ± 0.03	0.54 ± 0.03	104.30 ± 11.15	0.10 ± 0.01	
Unfavored	0.72 ± 0.02	0.50 ± 0.02	91.32 ± 12.93	0.128 ± 0.01	
*p* value	0.73	0.30	0.19	0.10	

## Supplementary Materials

The following are available online at https://www.mdpi.com/article/10.3390/insects12111047/s1. Supplementary S1, Detailed methods. Supplementary Figure S2, Proportion of eggs laid on each leaf of four trees (Tree B–E). Supplementary Figure S3, Correlations between number of eggs and visual information.
