# Supplementary material for: Swallowtail Butterflies Use Multiple Visual Cues to Select Oviposition Sites
Source: Insects. 2021 Nov 22;12(11):1047. doi: 10.3390/insects12111047 (PMC8622024; doi:10.3390/insects12111047)

Supplementary Materials

Swallowtail butterflies use multiple visual cues to select oviposition sites

Hiromi Nagaya, Finlay J Stewart, Michiyo Kinoshita

Laboratory of Neuroethology, SOKENDAI (The Graduate University for Advanced Studies), Shonan Village, Hayama 240-0193, Japan

e-mail: kinoshita_michiyo@soken.ac.jp

Supplementary S1 Detailed methods

The tracking procedure was similar to that described in Stewart et al. (2015) except that it was performed offline on recorded videos rather than in real-time. The projection matrices of the two cameras were calculated by manually identifying the positions of the arena vertices in the camera image, and then performing a least-squares fit. This represents a rectilinear approximation; radial distortion from the lenses was not accounted for.

For each video frame, we subtracted the previous frame. The resulting image was thresholded, and particle analysis was used to identify the largest connected region of supra-threshold pixels, corresponding to a rapidly changing part of the image, i.e. the flying butterfly. The centroid of this region, together with the projection matrix of the camera, define a ray in 3D space along which the animal must lie. This process was repeated for the other camera, and the frames were temporally aligned using a “clapperboard” cue recorded prior to the butterfly’s release into the cage. The individual 2D trajectories were smoothed by resampling with Gaussian convolution. For each timepoint, the mutually orthogonal line connecting the two cameras’ rays at their closest point was calculated, and if this was <30mm in length (i.e., the rays approximately intersected) then its midpoint was taken as the butterfly’s 3D position. If its length exceeded this value, a tracking error was assumed to have occurred and that frame was ignored.

Supplementary Figure S2. Proportion of eggs laid on each leaf of four trees (Tree B-E) In all cases, a few leaves were heavily favored for oviposition, although this tendency was not significant according to our metric. Average of proportion of laid eggs is shown as dashed line and number above the dashed line in each graph.

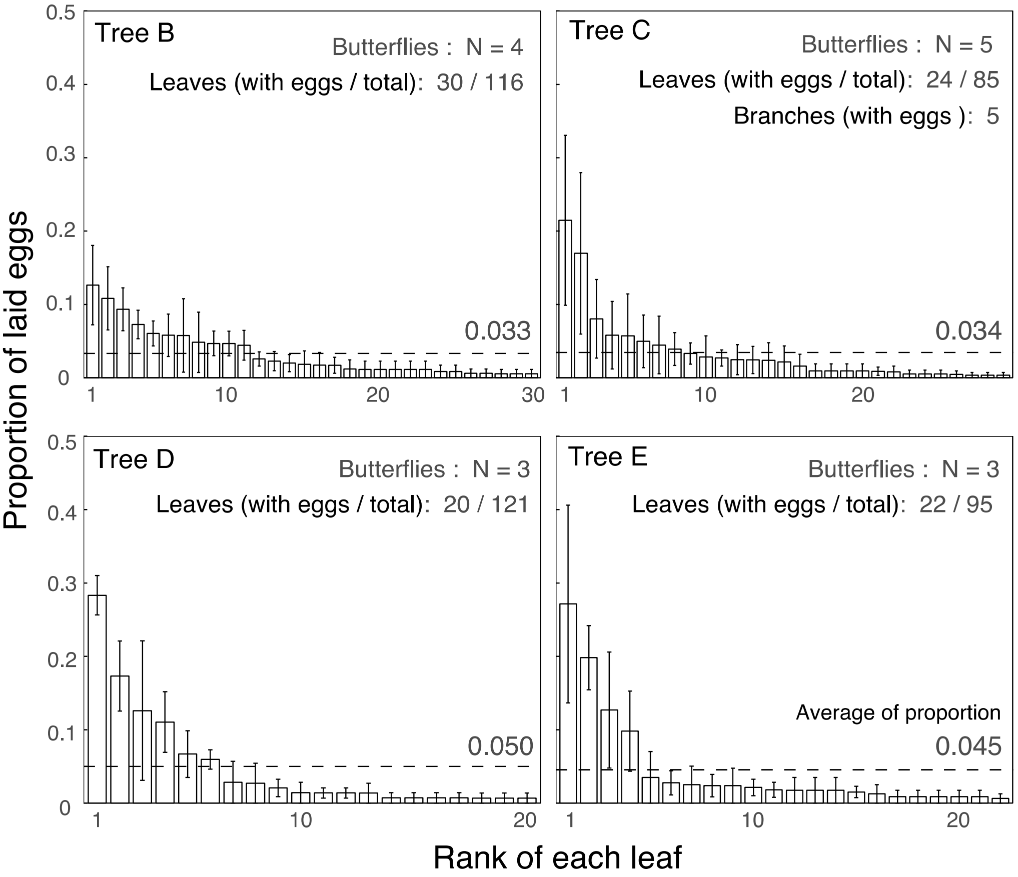

Supplementary Figure S3 Correlations between number of eggs and visual information. Brightness of leaves observed from directly above the tree is positively correlated with number of eggs (**A**. r = 0.45, p <0.01). Polarization degree measured from this position is also negatively correlated with number of eggs (**B.** r = -0.15, p < 0.05). Angle of polarization from both the right and left side of the tree (**C, D**) do not correlate with number of eggs (**C**: r = 0.07, p = 0.26, **D**: r = -0.05, p = 0.45).

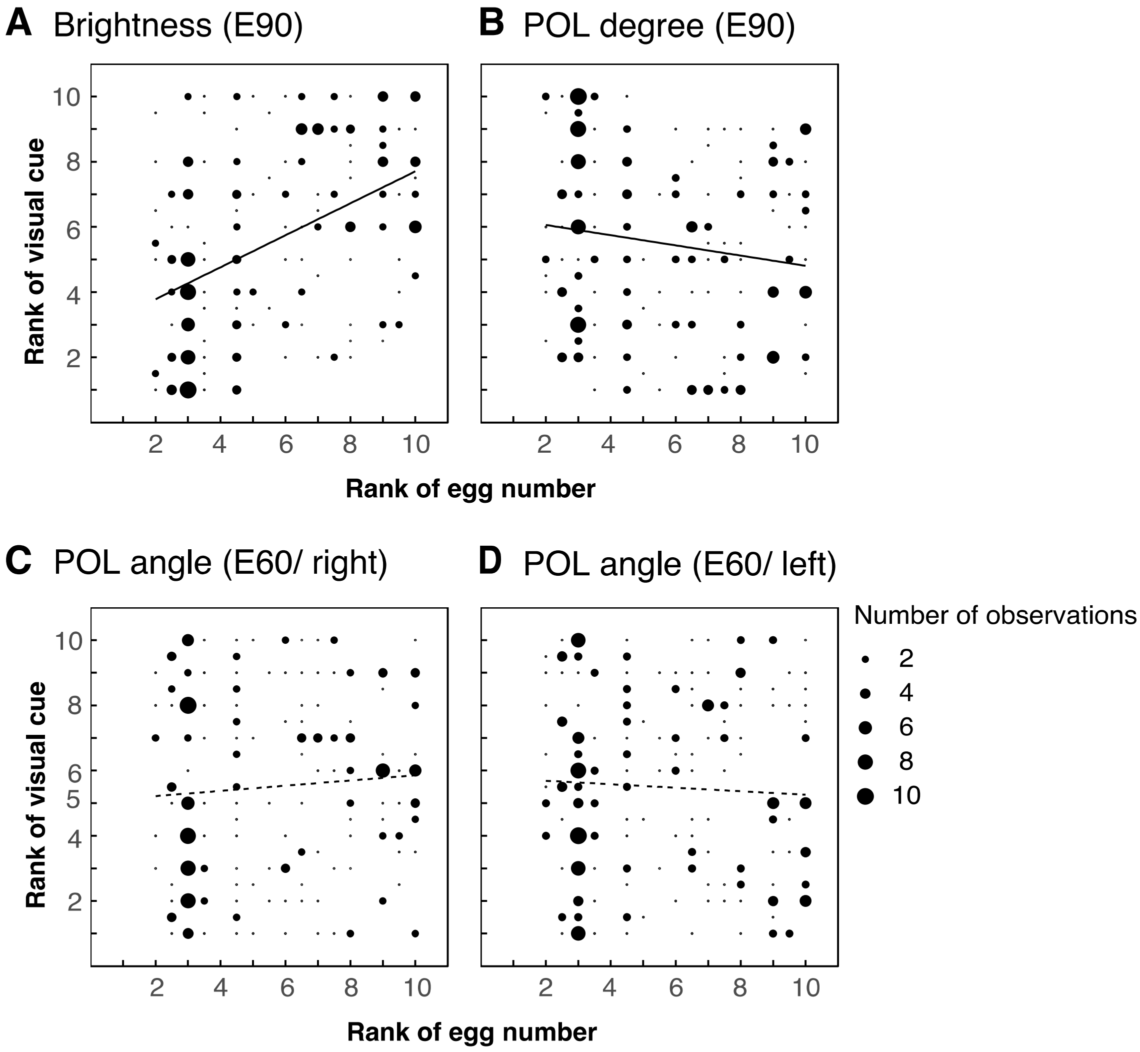

Supplement: Supplementary file 1 [file insects-12-01047-s001.zip › insects-1424131-supplementary.pdf]
